# Lighting Environment Optimization of Highway Tunnel Entrance Based on Simulation Research

**DOI:** 10.3390/ijerph16122195

**Published:** 2019-06-21

**Authors:** Yongqiang Zhang, Xi Zhuo, Wei Guo, Xiaoyu Wang, Zhenglu Zhao

**Affiliations:** 1 College of Automobile and Transport Engineering, Nanjing Forestry University, Nanjing 210037, China; zyqnjfu@sina.cn (Y.Z.); zyqnjfu2006@gmail.com (X.W.); zzl0319smu@yeah.net (Z.Z.); 2 College of Civil Engineering, Fuzhou University, Fuzhou 350108, China; 3 Ningbo University of Technology, Ningbo 315211, China; zyqseu9628@163.com

**Keywords:** tunnel entrance, lighting optimization, driving safety, simulation

## Abstract

At the entrance of a tunnel, reflection of sunlight from the surrounding environment and a lack of adequate lighting usually cause some vision problems. The purpose of this study is to optimize the lighting environment at the entrance of highway tunnels. Firstly, based on a highway tunnel in Zhejiang Province, the natural illumination intensity in different seasons and climate conditions inside and outside the tunnel entrance was analyzed by means of DIALux simulation software. Then the variation in illumination conditions with distance at the entrance of the tunnel was analyzed. Finally, based on the results above, this study proposes four solutions as follows: setting up a shading shed, auxiliary lighting facilities, decelerating reflective markings, and an adaptive dimming system.

## 1. Introduction

Health and safety issues associated with the traffic system, such as improving visibility for drivers, has attracted increasing attention in recent years due to the soaring rates of deaths, injuries, and disabilities resulting from road traffic accidents [1].

As the bottleneck section of a road network, there are significant traffic safety issues with highway tunnels. On the one hand, when the vehicle exits or enters the tunnel at a high speed, the cross-sectional light environment changes drastically and suddenly. The contradiction between this change and the limited adaptability of humans leads to the “black hole effect” [2,3]. Ahmad Mehri et al. [4] pointed out that the "black hole effect" at the tunnel entrance can lead to visual and psychological discomfort for drivers and can increase the risk of road traffic accidents. Due to the problems associated with illuminating the entrance zone of tunnels and to reduce the possibility of accidents in these areas, the International Commission on Illumination (CIE) [5] developed the standard CIE 88 in 1990. According to CIE 88-1990, the amount of light required at the entrance to a tunnel has been determined based on the average luminance in the 20° conical field of view to avoid the black-hole phenomenon [6].

On the other hand, vehicle drivers approaching a tunnel should have enough visibility into the tunnel from an appropriate distance that they are able to react in a timely manner if required [7,8]. For this reason, the CIE 88 defines a safe distance as the safe stopping distance (SSD). SSD is a distance that allows drivers to see any obstacle in a tunnel entrance and react appropriately to and stop in front of it. This distance is equal to the sum of the perception-reaction distance and the braking distance [9]. Narisada and Yoseoikawa [10] studied conditions that may interfere with the adaptation of a driver’s vision, and colleagues offered suggestions for adapting to these conditions.

Simulation and field testing are important means to study the change of tunnel entrance lighting. Adrian [11] presented a method for the subjective evaluation of lighting at the entrance of tunnels based on the CIE standard. Guo et al. [12] studied the effect of the external environment of highway tunnels on driving behavior, and the conclusion was drawn that rapid change of illumination at the entrance and exit of tunnels affects the driving safety of the road section; the safety design scheme for the entrance and exit of highway tunnels was proposed according to the driving speed. Hu et al. [13] studied drivers’ demand feature of driving vision at the tunnel entrance and the effect that illumination conditions have on drivers’ safety and comfort. Guo et al. [14] studied the aspect of driving vision in the segment of a tunnel at night and evaluated various solutions. Li et al. [15] put forward a solution using natural light as a light source in partially lit sections that have been divided. Mehri A. et al. [16] evaluated the safety of super-long tunnel lighting based on vision. Xue et al. [17] established a tunnel model using software and studied the scene sound of tunnel lighting caused by distributed lights in the tunnel. Cengiz M.S. [18] used computer software to determine the optimal solution for tunnel lighting design through analysis of tunnel lighting design.

In summary, researchers have studied the visual characteristics of the tunnel entrance drivers, and relatively little research on the distribution and variation of natural light intensity. In view of China’s special geographical and environmental climate conditions, this paper discusses the natural light intensity distribution and variation law at the entrance of the tunnel.

Given the functional effect on the luminous environment in a tunnel, the tunnel lighting guideline has explicitly stipulated that the effect of natural light on the driving environment at a tunnel entrance should be considered when designing tunnel illumination [19]. However, the locations and environments of highway tunnels are so varied that the effects of natural light change over seasons and time and long-term research on this topic remains relatively scarce. Therefore, based on the theoretical simulation of tunnel entrance natural light illumination, the research presented in this paper studies the law of illumination variation and the range of influence caused by natural light. By comparing the simulation results and the measured results, the variation law of natural light at tunnel entrances is discussed. The influence of natural light in different seasons, different climate models, and different time segments was analyzed. Finally, based on the results of the analyses outlined above, this study proposes four solutions as follows: setting up a shading shed, auxiliary lighting facilities, decelerating reflective markings, and an adaptive dimming system. The research conclusions can offer certain significance for optimizing the lighting environment of tunnel entrances and improving the driving safety of highway tunnels.

## 2. Theoretical Basis of Natural Light Illumination Simulation at Tunnel Entrances

The present research used the function of natural light analysis and calculation in simulation software, DIALux, based on the foundation of sky illumination distribution theory. DIALux can simulate, calculate, and analyze natural light intensity in any environment. It divides the vault of the sky into several illuminated areas parameterized according to the sunny day, cloudy day, and mixed day calculation models, and simulates and calculates the illumination intensity for various road surfaces by setting the sky illumination value according to different geographic positions, seasons, and specific moments. Simulation calculations were conducted according to three sky illumination distribution models, which are outlined below.

### 2.1. Sunny Day Sky Illumination Distribution Model

According to the standard full-sky lighting distribution model recommended by Kittler, the lighting calculation formula at any point in the sky is as follows [20]:

(1) $$L_{r}=\frac{(1-0.91e^{-0.32/\sin\gamma }-10e^{-3x-0.32/\sin\gamma }-0.45e^{-0.32/\sin\gamma }\cos^{2}X)\cdot L_{Z}}{1.184+2.74e^{-3Z_{s}}+0.1233\cos^{2}Z_{s}}$$

where *X* represents the shortest angular distance from point sky to the sun; *Z_s_* represents an angular distance from the to the vault of heaven; *L_γ_* represents sky element illumination, (cd/m^2^); *L_z_* indicates illumination of the vault of heaven, (cd/m^2^); *γ* represents an elevating angle of calculating point; and *Z* represents an angular distance from calculating point to vault of heaven, where *Z* = π / 2γ.

### 2.2. Cloudy Day Sky Illumination Distribution Model

The cloudy day sky illumination distribution model is determined according to the standard cloudy day sky illumination distribution model suggested by Moon and Spencer, and is calculated using Equation (2) below [20]:

(2) $$L_{r}=\frac{1}{3}(1+2\sin\gamma )\cdot L_{Z}=\frac{1}{3}\cdot (1+2\cos Z)\cdot L_{Z}$$

### 2.3. Mixed Sky Illumination Distribution Model

The mixed sky illumination distribution model encompasses the variation between a sunny day and cloudy day. The model is calculated according to the CIE guidelines using Equation (3) below [20]:

(3) $$L_{r}=\frac{f(X)\cdot \varphi (Z)}{f(Z_{s})\cdot \varphi (0)}\cdot L_{Z}$$

where *f*(*X*) represents the scattering exponential function, and *φ*(*Z*) represents an illumination gradient function, which is calculated using Equations (4) to (8).

(4) $$f(X)=1+c\cdot \left[\exp(dX)-\exp(d\frac{\pi }{2})\right]+e\cdot \cos^{2}X$$

(5) $$\varphi (Z)=\left(1+\alpha \cdot \left(\frac{b}{\cos Z}\right)\right)$$

(6) $$f(Z_{s})=1+c\cdot \left[\exp(dZ_{s})-\exp(d\frac{\pi }{2})\right]+e\cdot \cos^{2}Z_{s}$$

(7) $$\mathrm{and}\;u_{ik}\left(1\leq i\leq c,1\leq k\leq n\right)$$

(8) When, 0≤Z≤π/2.φ(0)=1+aexpb

substitute these results into Equation (3):

(9) $$L_{r}=\frac{\left(1+c\left[\exp(dX)-\exp(d\frac{\pi }{2})+e\cos^{2}X\right]\right)\cdot \left(1+a\cdot \exp\left(\frac{b}{\cos Z}\right)\right)}{\left(1+c\left[\exp(dX)-\exp(d\frac{\pi }{2})\right]+e\cos^{2}X\right)\cdot (1+a\exp b)}\cdot L_{Z}$$

where *a* and *b* represent illumination gradient parameters, and *c*, *d*, and e indicate scattering index functions. Sky illumination distribution calculating models under different conditions can be obtained by combining different parameters [20]. In fact, the mixed weather pattern is the most common situation, and the three weather patterns can be mutually transformed. We use equation (9) in the following simulation, to obtain the results.

## 3. Setting up a Natural Light Simulation Model of an Actual Tunnel in Zhejiang Province

The length of the sample tunnel in Zhejiang province is 2445 m. It is designed as a one-way, two-hole, and two-lane traffic tunnel, and the net width and net height of the entrance are 10.16 m and 6.98 m, respectively. A three-centered circular arch and bituminous pavement comprise the inner profile of the tunnel, and the entrance faces 45° northwest. In this study, the tunnel entrance lighting was the primary simulation object. In the process of using DIALux modeling, relevant parameters, such as space design, surface texture, geographical location, and calculation grid, were set.

### 3.1. Design Parameters of Tunnel Space Design

**Figure 1:**
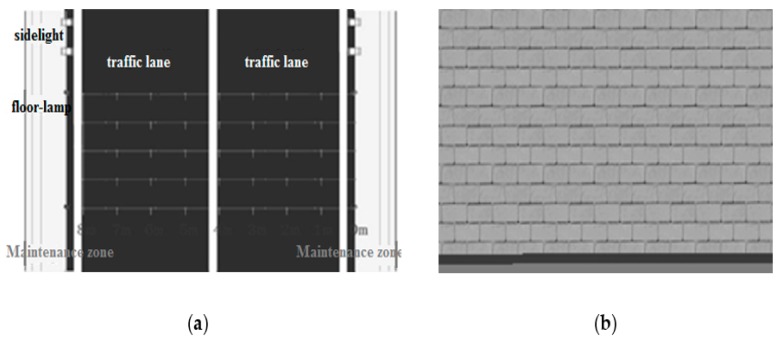
(**a**) Tunnel pavement simulation and (**b**) wall material simulation.

According to the measured tunnel design parameters, the feature points were input into the space design drawing. The design parameters included the total width of the tunnel (10.0 m), the net height of the lane (7.0 m), the total width of the maintenance lanes on both sides (8.0 m), the maintenance lane height (0.3 m), and the entrance length (100.0 m). Other parameters, such as longitudinal slope, horizontal line shape, and cross-sectional form, were consistent with the actual situation, as shown in Figure 1a.

### 3.2. Parameters of Tunnel Surface Texture

The corresponding texture properties of the ceiling, side walls, and pavements in the tunnel, including type and color, were used as the tunnel surface texture parameters. These properties have a decisive influence on reflectivity, transparency, and surface roughness. In accordance with the actual tunnel, the top of the simulated tunnel was painted with black fireproof paint, the pavement was bituminous pavement, and the side walls were covered with white ceramic tiles, 3 m from the ground, as shown in Figure 1b. In the model, the reflectivity of the ceiling, pavement, and side walls were 0.25, 0.2, and 0.5, respectively.

### 3.3. Parameters of Tunnel Geographic Position

According to the latitude and longitude of the actual tunnel, the direction facing the tunnel entrance, and the location selected by the tunnel, the tunnel geographical parameters are: 29°29′ N 120°88′E, and the southeast direction is 45°.

### 3.4. Computational Grid of Pavement Illumination

**Figure 2:**
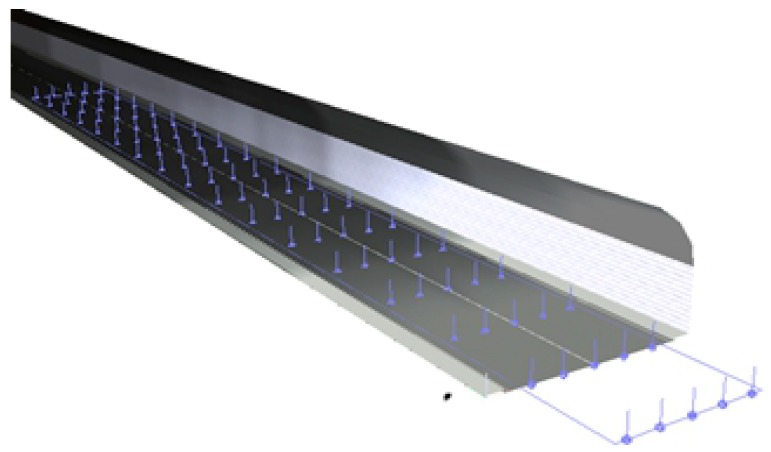
Division of pavement illumination simulation grid.

**Figure 3:**
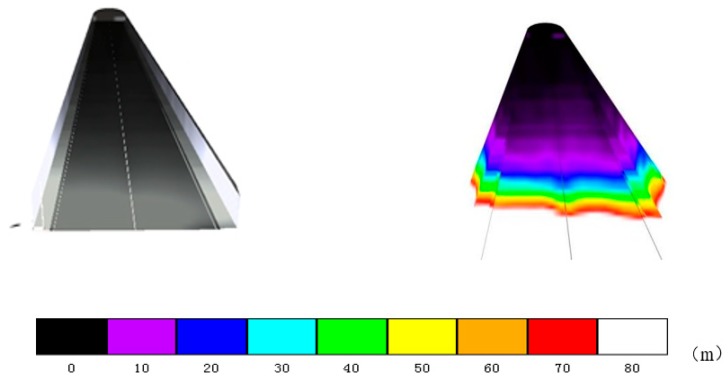
Natural light simulation result.

The section of road ranging from 40 m outside the tunnel entrance to 60 m inside the tunnel entrance was selected as the computational zone, and the zone was divided into computational grids according to 12 lengthways computational points (distance between points was 5 m), and 5 transverse computational points (distance between points was 0.75 m). The division of pavement illumination simulation computational grid and its simulating effect are presented in Figure 2 and Figure 3. After the parameters above were set, the simulation computation of pavement illumination was carried out for different seasons, weather conditions, and moments.

## 4. Simulation Computation Results of Natural Light Variation at Tunnel Entrances

### 4.1. Simulation Computation of Pavement Illumination at Tunnel Entrances

**Figure 4:**
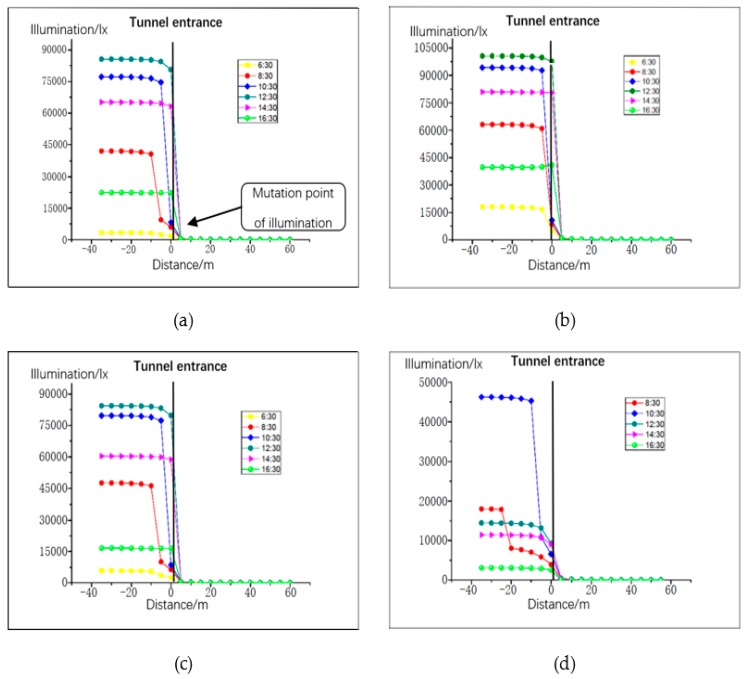
Road illumination for clear weather pattern: (**a**) vernal equinox, (**b**) summer solstice, (**c**) autumn equinox, and (**d**) winter solstice.

**Figure 5:**
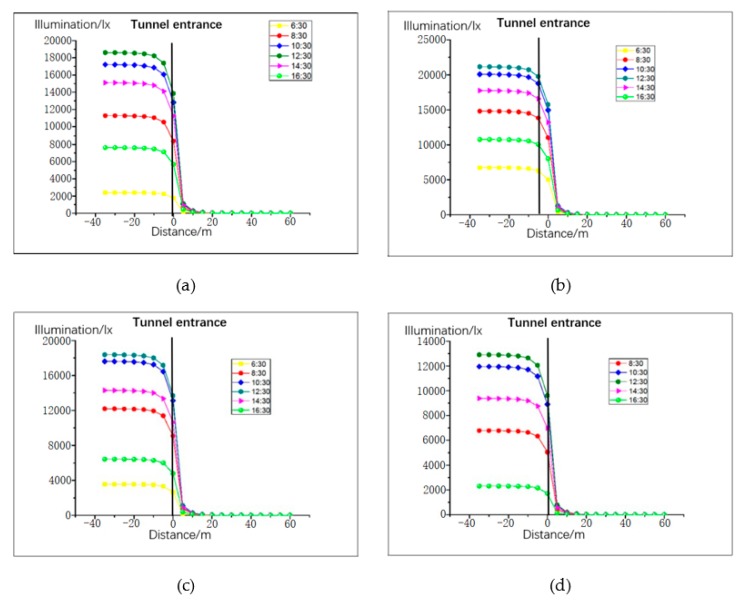
Road illumination for cloudy weather pattern: (**a**) vernal equinox, (**b**) summer solstice, (**c**) autumn equinox, and (**d**) winter solstice.

**Figure 6:**
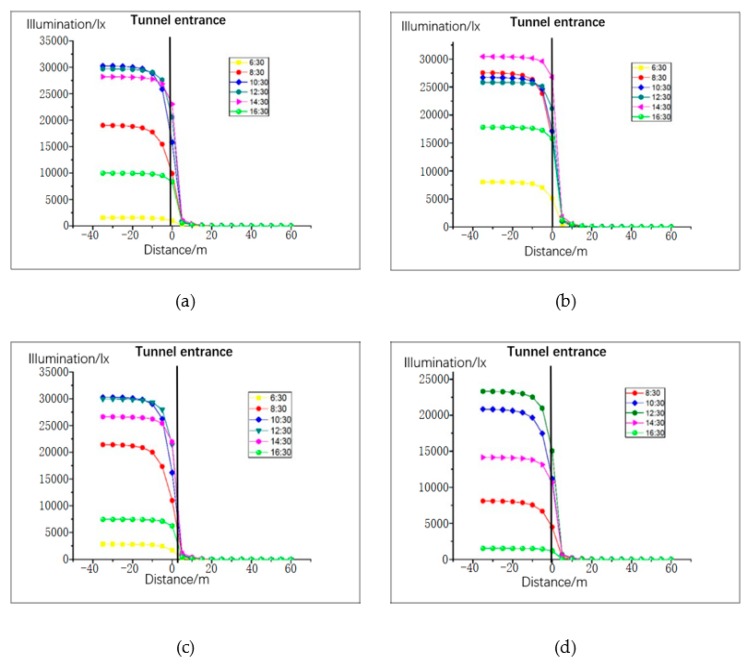
Road illumination for mixed weather pattern: (**a**) vernal equinox, (**b**) summer solstice, (**c**) autumn equinox, and (**d**) winter solstice.

The distance between the left and right lanes in the tunnel is relatively small, and the illumination distribution law is basically equal. Therefore, the distribution of road illumination was simulated and analyzed for the right lane only. Lighting distribution is greatly influenced by season, time, and climatic conditions. Thus, the trend of natural light was simulated between 06:30 and 17:30 on the days of spring equinox, summer solstice, autumn equinox, and winter solstice. At 06:30 on the winter solstice, the amount of light was basically 0, so we chose to start at 06:30 in the morning. The climatic conditions were clear, cloudy, and mixed days, as presented in Figure 4, Figure 5 and Figure 6. The abscissa is the distance from the tunnel entrance, the left side of coordinate 0 represents the outside of the tunnel, and the right side of coordinate 0 represents the inside of the tunnel. The ordinate is the illumination intensity (lx).

### 4.2. Simulation Result Analysis of Tunnel Entrance Pavement

From the simulation results presented in Figure 4, Figure 5 and Figure 6, the following conclusions were drawn:

- The illumination of the road outside the tunnel is not obvious. From the tunnel entrance (−40m), the maximum change in the four solar terms a year was 2141 (lx) (8:30 sunny day on summer solstice). The rate of change was 12%.
- With the change of the distance to the tunnel entrance, the illumination of the whole lane changes in varying degrees. At a distance of −5 m to 5 m, the light changes most intensely. At 12:30 in summer, when the distance changed from −5 m to 5 m, the light intensity decreased from 97671 (lx) to 282 (lx), with a change rate of 99.71%. Even on cloudy winter days, when the distance changed from −5 m to 5 m, the illumination changed from 96111 (lx) to 207 (lx), with a change rate of 97.84%.
- On sunny days, the light intensity from 12:30 to 14:30 was greater than at other times before 12:00 and later in the afternoon, and the correlation between cloudy days and mixed days was lower.
- When the distance between the tunnel interior and the tunnel entrance was greater than 10 m, the change trend of natural light clearly decreased; when the distance was greater than 20 m, the maximum value of natural light in the four seasons was only 59 (lx). Thus, the influence of natural light on the tunnel environment can be neglected.

### 4.3. Comparison of Pavement Illumination Simulation and Actual Measurement Results

In order to verify the objectivity and accuracy of the simulation results of road lighting, an illuminometer was used to measure the illumination of the tunnel entrance. According to the data of past traffic accidents at the tunnel entrance, we chose the accident-prone time interval (11:40–15:30) for measurement. Three illuminometers were installed. One was installed 5 m outside the tunnel entrance and measurement was recorded once per second. The other two illuminometers measured the road illumination at 0 m and 5 m inside the tunnel entrance. Each point was measured for 180–240 minutes.

Mixed weather is the most common in different seasons of the year. Therefore, taking the mixed weather (14:30) data as an example, the measured results of the illuminance change rate were compared with the simulation results, as shown in Table 1.

From Table 1, the following conclusions can be drawn:

1. The measured and simulated data (rate of change) outside the tunnel (−5 m) to the entrance of the tunnel are relatively close, which proves the correctness of the simulation results.
2. The measured and simulated data in the tunnel entrance (5 m) are quite different. This deviation is due to the fact that, after entering the tunnel entrance, the internal light is reflected by multiple reflections of different materials on the side wall of the tunnel and the air quality inside the tunnel.

### 4.4. Improvement Measures of the Lighting Environment at Tunnel Entrances

1. Shading shed at tunnel entrances

**Figure 7:**
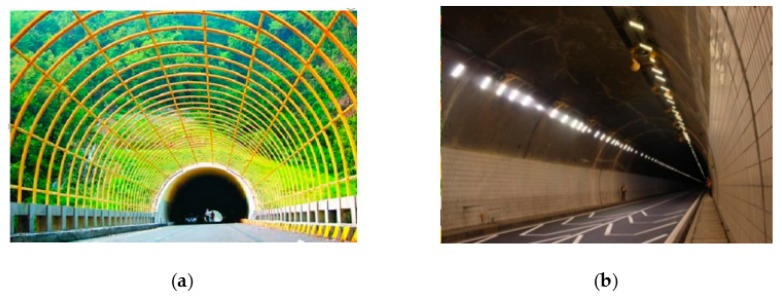
(**a**) Shading shed and (**b**) auxiliary strengthen lighting and reflective road markings.

A shading shed is a type of light-reducing shed that follows the same shape as the tunnel entrance. The shading shed is generally 50 m in length and is placed at a certain distance, as illustrated in Figure 7a. A shading shed embedded in a steel frame can reduce the traffic interference and lighting in the shadow section, and allow the driver to adapt more easily to the changes of the tunnel light environment.

2. Auxiliary-strengthened lighting at tunnel entrances

The high probability of large trucks driving through highway tunnels and severe haze at tunnel entrances can cause visibility issues for drivers inside tunnels. In order to reduce the “black hole effect” at tunnel entrances, the proposed study recommends that low color temperature tunnel lamps be arranged to assist lighting at both sides of the tunnel entrance, as shown in Figure 7b.

3. Road markings with decelerated reflective light at tunnel entrances

Specially-designed road markings, set within the range of 200 m outside the tunnel to 100 m inside the tunnel, are helpful for traffic safety, as illustrated in Figure 7b. As a vehicle enters the front lane of the tunnel entrance, these markings can cause an illusion that the lane is narrowing and the road is sinking, thereby causing the driver to reduce their driving speed.

4. Adaptive light dimming control system at tunnel entrances

The adaptive dimming system can effectively control the illumination value of a tunnel entrance according to real-time illumination observation value, traffic flow, average speed outside the tunnel, and road illumination value inside the tunnel, as well as reduce the influence of illumination change on drivers and improve driving safety.

In summary, the improvement of lighting environment at tunnel entrance is mainly considered from two aspects: inside and outside the tunnel entrance. Outside the tunnel entrance, the setting of a shading shed can effectively improve the driver’s eye adaptability. By adding auxiliary lighting to the tunnel, the driver can better adapt to the light and dark changes in the tunnel, but this increases operating costs. Both internal and external methods have improved the tunnel entrance lighting environment and ensured driving safety.

## 5. Conclusions and Prospect

In the research presented in this paper, a calculation model of road lighting at the entrance of a highway tunnel in Zhejiang province was established by using simulation software. By comparing the simulation results with the measured results, the variation law of natural light at the entrance of a highway tunnel was discussed. The influence of natural light on the lighting environment of the tunnel entrance in different seasons, climate patterns, and times was then analyzed. The results indicate that the change of natural illumination is the strongest in the range of 5 m from the tunnel entrance to the tunnel entrance, and the change in range of natural illumination above 5 m from the tunnel entrance is clearly reduced. Different geographical locations have different characteristics of light and climate. Therefore, further long-term and more detailed studies are needed to analyze the variation in natural light intensity under different geographical locations and climatic conditions. Finally, based on the results presented above, this study proposed four possible solutions: the installation of shading sheds, auxiliary lighting facilities, decelerating reflective markings, and adaptive dimming systems. The research results are significant for optimizing the lighting environment of tunnel entrances and improving traffic safety.

## Figures and Tables

**Table 1:** Comparisons of measured and simulated calculation rate of change results (mixed weather pattern).

Distance (m)	Vernal Equinox		Summer Solstice		Autumn Equinox		Winter Solstice	
	Me%	Si%	Me%	Si%	Me%	Si%	Me%	Si%
(−5)−0	23.86	25.56	16.25	15.83	21.87	23.4	17.34	16.82
0–5	85.63	96.80	82.77	99.28	80.35	99.96	76.39	96.65

NOTE: Me = Measurement; Si = Simulation.
